# Correction to: 3D bioprinted tumor model: a prompt and convenient platform for overcoming immunotherapy resistance by recapitulating the tumor microenvironment

**DOI:** 10.1007/s13402-024-00952-8

**Published:** 2024-04-30

**Authors:** Zhanyi Zhang, Xuebo Chen, Sujie Gao, Xuedong Fang, Shengnan Ren

**Affiliations:** 1 Bethune Third Clinical Medical College, Jilin University, 130021 Changchun, China; 2 Department of Gastrointestinal, Colorectal and Anal Surgery, China-Japan Union Hospital of Jilin University, NO. 126, Xiantai Street, 130033 Changchun, China; 3 Department of Anesthesiology, China-Japan Union Hospital of Jilin University, 130033 Changchun, China; 4 Department of Breast Surgery, Peking University Cancer Hospital Yunnan, Yunnan Cancer Hospital, The Third Affiliated Hospital of Kunming Medical University, NO. 519, Kunzhou Street, 650118 Kunming, China

**Correction to: Cellular Oncology**

10.1007/s13402-024-00935-9

In this article the author’s name Zhanyi Zhang was incorrectly written as Zhangyi Zhang.

In addition, Supplementary Information was wrongly included.

The original article has been corrected. 

